# Possible linkage between asymmetry of atmospheric meridional circulation and tropical cyclones in the Central Pacific during El Niño years

**DOI:** 10.1371/journal.pone.0259599

**Published:** 2021-11-05

**Authors:** Tao Li, Fajin Chen, Shuwen Zhang, Xiaoli Feng, Weiqiang Zeng

**Affiliations:** 1 College of Ocean and Meteorology, Guangdong Ocean University, Zhanjiang, Guangdong, China; 2 Institute of Marine Science, Shantou University, Shantou, Guangdong, China; Guangzhou University, CHINA

## Abstract

The El Niño–Southern Oscillation is one of the most important drivers of climate change on Earth, and is characterised by warmer (El Niño) or colder (La Niña) ocean surface temperatures in the equatorial Pacific. Tropical cyclones (TCs) and meridional circulation are the most influential weather events and climate phenomena, respectively. However, the link between TCs and meridional circulation anomalies (MCA) during El Niño years is unclear. Therefore, we calculated the accumulated cyclone energy index of TCs and the mass stream function of MCA from 1980 to 2018. Our results showed that TCs were closely related to the asymmetry of the MCA in the Central Pacific during El Niño years. An updraft anomaly in the North Pacific was found, which affected the response of MCA to El Niño from May to October during El Niño years. Therefore, the MCA intensity difference between the North and South Pacific increased, and the asymmetry was strengthened. This phenomenon may be strengthened by the combined effects of the equatorial westerly wind, relative vorticity, and warm ocean surfaces, which are controlled by El Niño. The equatorial westerly wind produces positive shear north of the equator, which increases the relative vorticity. The increase in relative vorticity is accompanied by a monsoon trough, leading to increased precipitation and updrafts. The background of the relative vorticity, updraft, and monsoon trough may be conducive to the generation and development of TCs. Our results prove that the possible link between TCs and the asymmetry of the MCA during El Niño years is derived from the combined effect of the equatorial westerly wind, relative vorticity, and warm ocean surfaces, thus providing a partial explanation for the link between TCs and the MCA.

## Introduction

El Niño and La Niña (known as the El Niño–Southern Oscillation (ENSO)) represent the strongest interannual fluctuation of the global climate system. Due to changes in the atmosphere and ocean circulation, ENSO can affect the global atmosphere, ocean/terrestrial ecosystems, fisheries, agriculture, and human activities [1]. Extreme El Niño events have been severely destructive. For example, the extreme El Niño of 2015–16 caused the longest global coral bleaching event on record [2], which is estimated to require more than 10 years to recover. The extreme El Niño of 1997–98 led to reduced rainfall and widespread forest fires across Indonesia. Smog emitted from the fires covered six countries and threatened the health of 70 million people [3]. Moreover, the negative impact of extreme El Niño events is even more significant in poorer areas. For instance, Africa’s fragile agricultural sector was impacted by severe floods and droughts in 2015, and cholera, malaria, and hunger threatened 60 million people [4]. Due to these destructive effects, El Niño impacts on the atmosphere is an important topic in meteorological studies.

Tropical cyclones (TCs) are one of the most destructive natural disasters [5] and have significant societal and economic impacts worldwide [6]. For example, in 2005, Hurricane Katrina was the largest meteorological natural disaster recorded in the United States, causing a loss of 149 billion US dollars and leaving millions of people homeless. Storm surges caused by extreme TCs are even more deadly [7]. In 1970, Cyclone Bora brought a storm surge with a height of 9.1 m to Bangladesh, causing approximately 300 000 deaths. Today, more than 250 million people live in locations below the highest storm-surge water level; hence, these regions are under the constant threat of death and destruction from TCs-induced storm surges. There is currently a lack of consensus on how global warming affects TCs [8], but the intensity of TCs has increased over the past 40 years [9], and mounting evidence suggests that the frequency of stronger TCs has increased significantly [10–12]. As TCs are sensitive to tropical climate conditions [13], ENSO may have a significant impact on global TCs activities [14], and the impact may increase in the context of future global warming [15]. Owing to the negative effects of TCs, the relationship between TCs and ENSO is also an important issue in meteorology.

The low-latitude meridional circulation (MC), also called the Hadley circulation, is a thermally driven MC in tropical regions. MCs play an important role in balancing the mass, energy, and angular momentum of the atmosphere between the two hemispheres [16]. Therefore, changes in the MC can significantly affect global climate change [17]. The sea surface temperature anomaly (SSTA) in the tropics has an important influence on the meridional circulation anomaly (MCA). Research has shown that the meridional tropical sea temperature gradient can drive significant MC and water vapour convergence in the lower troposphere [18], resulting in MC ascending branches at the maximum latitude of the SSTA. A simple single-layer model reveals that the zonal average low-level meridional current (the lower branch of the MC) is driven by the zonal average meridional SST gradient [19]. In addition, the meridional gradient of atmospheric heating is important for the formation of MC structure [20]. Recent studies have identified a linear relationship between the symmetry of the MCA and zonal average SSTA, which suggests that the zonal average of the equatorial symmetric (asymmetric) SSTA can lead to an equatorial symmetric (asymmetric) MCA [21, 22]. Under this linear constraint, El Niño is an important forcing of the MCA symmetry because it has an equatorially symmetrical zonal average SSTA [21, 23]. However, some researchers have identified a close link between El Niño and MCA asymmetry [24, 25]. The MCA in the West and Central Pacific largely determines the interannual variability of the boreal summer southern Hadley cell in the first leading mode [25]. The MCA asymmetry is related to the generation of TCs in the West and Central Pacific, and some large-scale environmental factors (vorticity, vertical velocity, etc.,) related to the asymmetry of the MCA affect the generation and development of TCs in the West and Central Pacific [26]. As the MCA also has an asymmetric response to symmetric El Niño forcing, it is unclear whether this asymmetric response is also related to the monsoon trough (MT) and TCs. Previous studies have assessed the MCA response to El Niño based on the inter-annual changes of seasonal average or annual average MCA, but they did not include changes on the yearly timescale [26, 27]. It is, therefore, necessary to assess the monthly changes in the MCA during El Niño years to further elucidate the impact of El Niño on the atmosphere. In this study, we used the latest generation of atmospheric reanalysis data (ERA5) to calculate the mass stream function (MSF) of the MCA. We also used the International Best Track Archive for Climate Stewardship (IBTrACS) of TCs to calculate the TCs accumulated cyclone energy (ACE) index and explore the possible connection between the MCA and TCs during El Niño events.

## Data and methods

### Data

The maximum 1-min sustained wind speed and central minimum pressure of TCs in the North Pacific Ocean from 1980 to 2018 were obtained from the IBTrACS (available online at https://www.ncdc.noaa.gov/ibtracs/) [28].

The Oceanic Niño Index (ONI) was used to describe ENSO events in the present study. ONI is calculated based on the 3-month moving average of the SSTA in the NINO-3.4 region (5°N–5°S, 120°W–170°W). ONI data from 1980 to 2018 were obtained from the National Oceanic Atmospheric Administration (NOAA) (available online at https://origin.cpc.ncep.noaa.gov/products/analysis_monitoring/ensostuff/ONI_v5.php).

The monthly average global sea surface temperature, horizontal wind, vertical wind, total precipitation, sea level pressure, and relative vorticity (0.25° × 0.25°) from 1980 to 2018 were derived from the fifth-generation reanalysis data of the European Centre for Medium-Range Weather Forecasts (ERA5, https://cds.climate.copernicus.eu, DOI: 10.24381/cds.6860a573).

### Methods

#### Selection criteria for El Niño events

The selection criteria for El Niño events were based on the NOAA standards (https://ggweather.com/enso/oni.htm). The events were defined as five consecutive overlapping 3-month periods at or above the +0.5 anomaly for warm (El Niño) events, and at or below the -0.5 anomaly for cold (La Niña) events. The threshold was further categorised into weak (with a 0.5 to 0.9 SSTA), moderate (1.0 to 1.4), strong (1.5 to 1.9), and very strong (≥ 2.0) events. In this study, an event must have equalled or exceeded the threshold for at least three consecutive overlapping 3-month periods for it to be categorised as weak, moderate, strong, or very strong.

For our study, we selected eight El Niño events (Table 1) with moderate intensity or above between 1980 and 2018 (1982–1983, 1986–1987, 1991–1992, 1994–1995, 1997–1998, 2002–2003, 2009–2010, and 2015–2016). The 1987–88 El Niño event was excluded because it was a continuation of the 1986–87 El Niño event. We focused particularly on the development stage of El Niño. El Niño’s developing year is defined as the year in which El Niño transitions from weak to strong, whereas El Niño’s decaying year is defined as the year in which El Niño transitions from strong to weak.

**Table 1:** El Niño of different intensities from 1980 to 2018.

El Niño			
Weak (0.5–0.9)	Moderate (1.0–1.4)	Strong (1.5–1.9)	Very Strong (> = 2.0)
2004–05 (0.7)	1986–87 (1.2)	1987–88 (1.5)	1982–83 (2.2)
2006–07 (0.8)	1994–95 (1.0)	1991–92 (1.5)	1997–98 (2.3)
2014–15 (0.5)	2002–03 (1.1)		2015–16 (2.5)
2018–19 (0.8)	2009–10 (1.4)		

Excerpt from NOAA (https://ggweather.com/enso/oni.htm). The numbers in parentheses indicate that the ONI had equalled or exceeded the threshold for at least three consecutive overlapping 3-month periods.

#### Accumulated Cyclone Energy (ACE) index

ACE is an index used to measure the activity of TCs. It combines the number of TCs systems, their duration, and their intensity. It is calculated by squaring the maximum sustained surface wind in the system every 6 hours that the TCs are a classified as a “Named Storm”, and then summing it up for the period. The ACE index was calculated using Eq (1):

$$\text{ACE}=10^{-4}\sum {\text{v}}_{\text{max}}^{2},$$ (1)

where v_max_ is the estimated sustained wind speed in knots).

#### Z-score normalisation

To conveniently compare the variables and results, it is necessary to normalise the data. The normalised variable can be calculated using Eq (2):

$${\text{x}}^{*}=\frac{\left(\text{x}-\mu \right)}{\sigma },$$ (2)

where μ and σ are the mean and standard deviation of the sample data, respectively.

#### Mass Stream Function (MSF)

The meridional-vertical MSF is an important index for describing MC [29]. In spherical coordinates, the MSF (Ψ) at each pressure level (p) and latitude (*θ*) can be expressed as:

$$\Psi (\theta ,\text{p})=\frac{2\pi \;\text{acos}\;\theta }{\text{g}}\int_{0}^{\text{p}}\bar{\text{V}}(\theta ,\text{p})\cdot \text{dp},$$ (3)

where a is the radius of the Earth, g is the acceleration due to gravity, and $\bar{\text{V}}$ is the regional average meridional wind speed in the selected region. We define the average MSF in the South Pacific (0°–30°S, 140°E–140°W, 10-hPa–1000-hPa) as the SMSF, and SMSF * -1 was defined as a negative SMSF.

#### Relative vorticity in the North Pacific (NRV)

NRV is defined as the average relative vorticity at 850-hPa in the North Pacific (0°–20°N, 140°E–140°W). The monthly average global relative vorticity (0.25° × 0.25°) from 1980 to 2018 was derived from the ERA5.

#### Equatorial Westerly Wind (EWW)

EWW is defined as the zonal average wind speed at 850-hPa in the equatorial region (5°S–5°N, 140°E–140°W). The monthly average global wind speed (0.25° × 0.25°) from 1980 to 2018 was derived from ERA5.

#### Data pre-processing

All monthly datasets were pre-processed using the 3-month moving average. All variables in the research were abnormal quantities. We calculated monthly climatic averages from 1980 to 2018, and the anomalous field was obtained by subtracting the monthly climatic averages.

## Results and discussion

### Meridional circulation anomaly response to El Niño

Studies have investigated the MCA in the North and South Pacific during ENSO since 1996 [29]. Their findings suggest that the MCAs between 0° and 30°N in the North Pacific and between 0° and 30°S in the South Pacific were closely related to ENSO events [29]. Recent research suggests that the SSTA in the tropics has an important impact on the MCA [21, 22]. A linear relationship exists between the symmetry of the MCA and the zonal average SSTA, which suggests that the zonal average equatorial symmetric (asymmetric) SSTA can lead to an equatorial symmetric (asymmetric) MCA. The SSTA in the equatorial region during the ENSO event was approximately symmetric about the equator. The MCA in the North and South Pacific during ENSO can be regarded as the direct thermal circulation released by equatorial SSTA energy [30].

**Fig 1:**
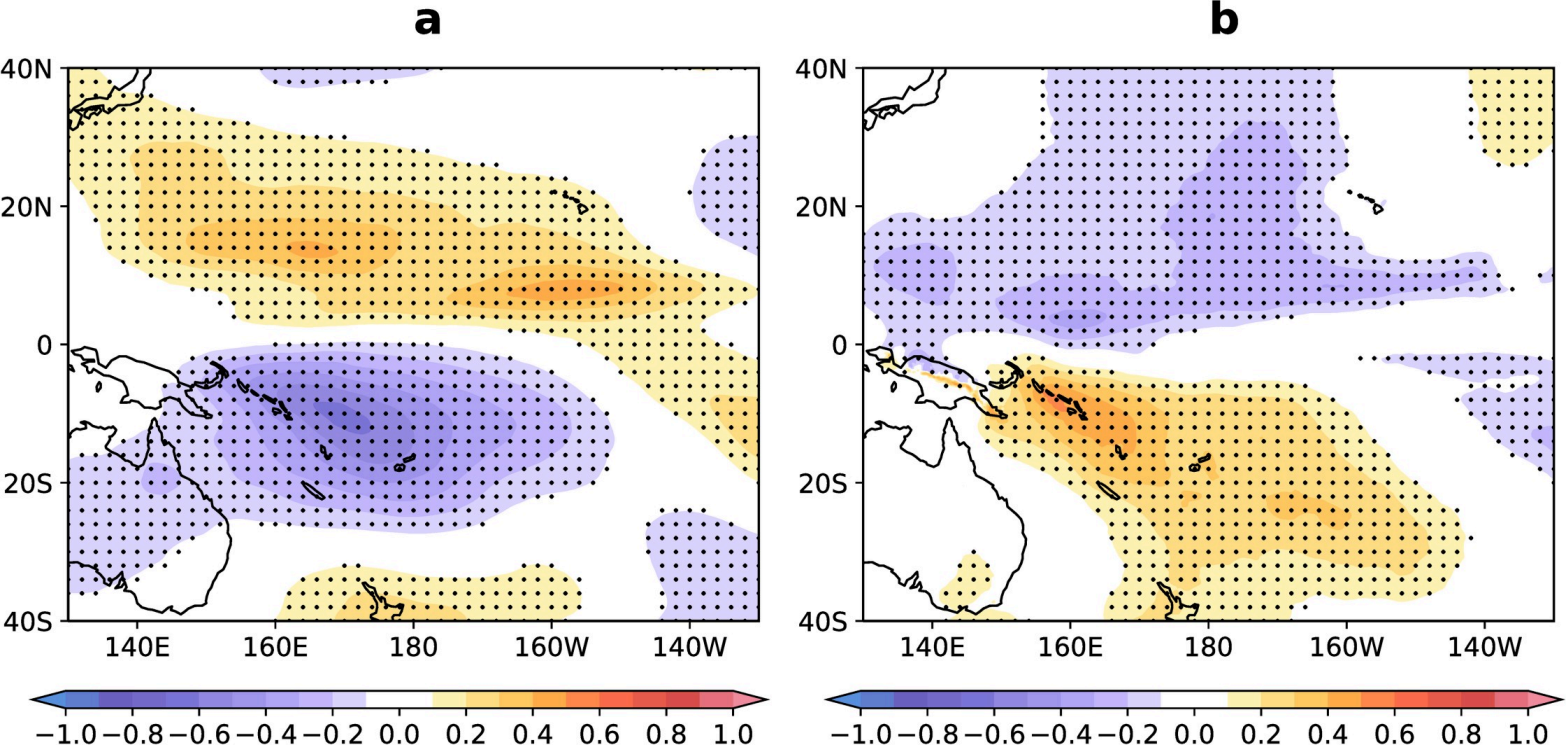
MCA response to El Niño. The shaded area indicates the correlation coefficient between the ONI (°C) and the meridional wind speed anomaly (m s^-1^) at 200-hPa (a) and 850-hPa (b). The stippled regions indicate a significance of > 95% confidence level using the Student’s t-test.

To identify the region in which the MCA responds to El Niño in the Pacific and extend the connection between the MCA and TCs during El Niño years, we calculated a linear regression between ONI and the meridional wind anomaly at 200-hPa and 850-hPa in the Pacific, and the results are presented in Fig 1. A positive (negative) correlation indicates a northward (southward) migration of the wind anomaly with the development of El Niño. The airflow of the MCA flows to the North and South Pacific at 200-hPa (Fig 1A) and then back to the equator at 850-hPa (Fig 1B) during El Niño years. The MCA response to El Niño at 200-hPa and 850-hPa (Fig 1A and 1B) exhibits obvious equatorial symmetry, which is intuitive and consistent with previous research [21, 23]. The SSTA occurs in the equator and has an equatorial symmetric structure, so it is closely related to the equatorial symmetric MCA during El Niño years.

The Central Pacific generally lies between 180°W–140°W. Our research on the MCA includes both the Central Pacific (180°W–140°W) and the south-eastern West North Pacific (WNP, 140°E–180°E). The areas of significant MCA response to El Niño were mainly concentrated within 140°E–140°W (Fig 1A and 1B). In the following sections, we collectively refer to the above two regions as the Central Pacific (140°E–140°W).

**Fig 2:**
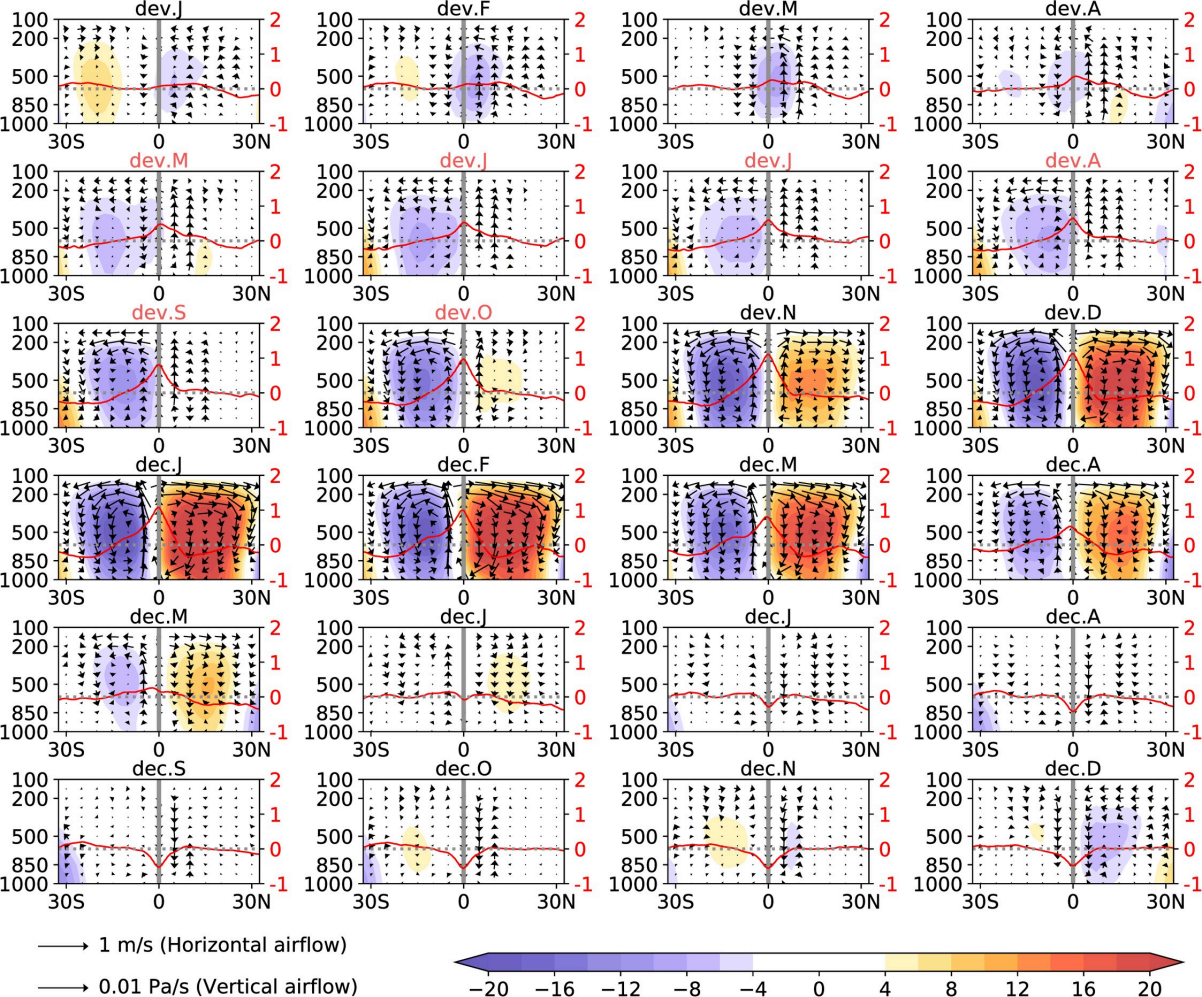
Composite MCA in the Central Pacific (140°E–140°W) during El Niño years. The shading indicates the average zonal MSF of eight El Niño events (unit: 10^10^ kg s^-1^). The purple area indicates counter-clockwise movement, and the red or yellow areas indicate clockwise movements. The arrow indicates the magnitude and direction of the wind speed (unit of horizontal airflow: m s^-1^; unit of vertical airflow: 10^−2^ Pa s^-1^). The red line indicates the zonal average (140°E–140°W) SSTA (unit: °C). The grey line indicates the equator. Title dev. J-D indicates January–December of the El Niño developing year; dec. J-D indicates January–December of the El Niño decaying year. MCA asymmetry mainly occurs during May–October of the El Niño developing year, which are marked in red.

To research the monthly MCA changes during El Niño years, 8 El Niño events from 1980 to 2018 were presented: 1982–83, 1986–87, 1991–92, 1994–95, 1997–98, 2002–03, 2009–10, 2015–16. As each El Niño event has obvious differences, analysing each one individually will cause confusion and fail to identify the commonality of the MCA asymmetry. Therefore, we first averaged the MCA of the eight El Niño events to better investigate the asymmetry of the MCA during El Niño years. We used the MSF to describe the MCA, as the zonal average MSF is an important indicator for describing MC [29]. After averaging the MSF of the MCA, we observed the MCA during El Niño years, and the results are presented in Fig 2. SSTA is concentrated in the equator with only one peak, and is approximately symmetrical from May to October during the El Niño developing year (Fig 2, dev5–dev10, marked in red). Driven by the SSTA, only one MCA in the South Pacific was formed. The airflow of the MCA rises in the equator and sinks near 20°S. The intensity of the MCA in the North and South Pacific showed obvious asymmetry. This phenomenon may be influenced by the combined effect of the EWW, NRV, and a warm ocean surface, which is further discussed in the following sections.

We observed an updraft anomaly (represented by the updraft in the following) between 0°–20°N that coexists with the asymmetry of the MCA from May to October during the El Niño developing year. This specific airflow may lead to the asymmetry of MCA. An equatorially symmetric SSTA can lead to an equatorially symmetric MCA during El Niño years [21, 22]. The occurrence of an updraft between 0° and 20°N during El Niño development can block the formation of the MCA in the North Pacific, as the updraft can block the sinking branch of the MCA in the North Pacific. Therefore, the MCA in the North Pacific may be blocked by the updraft between 0°–20°N and either develops slowly or ceases to generate, while the rapid development of MCA in the South Pacific leads to the asymmetry of the MCA from May to October during the developing year. The existence of this mechanism requires further verification of the physical mechanisms and models, but it can still be verified via phenomenon observations and statistics.

### Updraft affects meridional circulation anomaly

**Fig 3:**
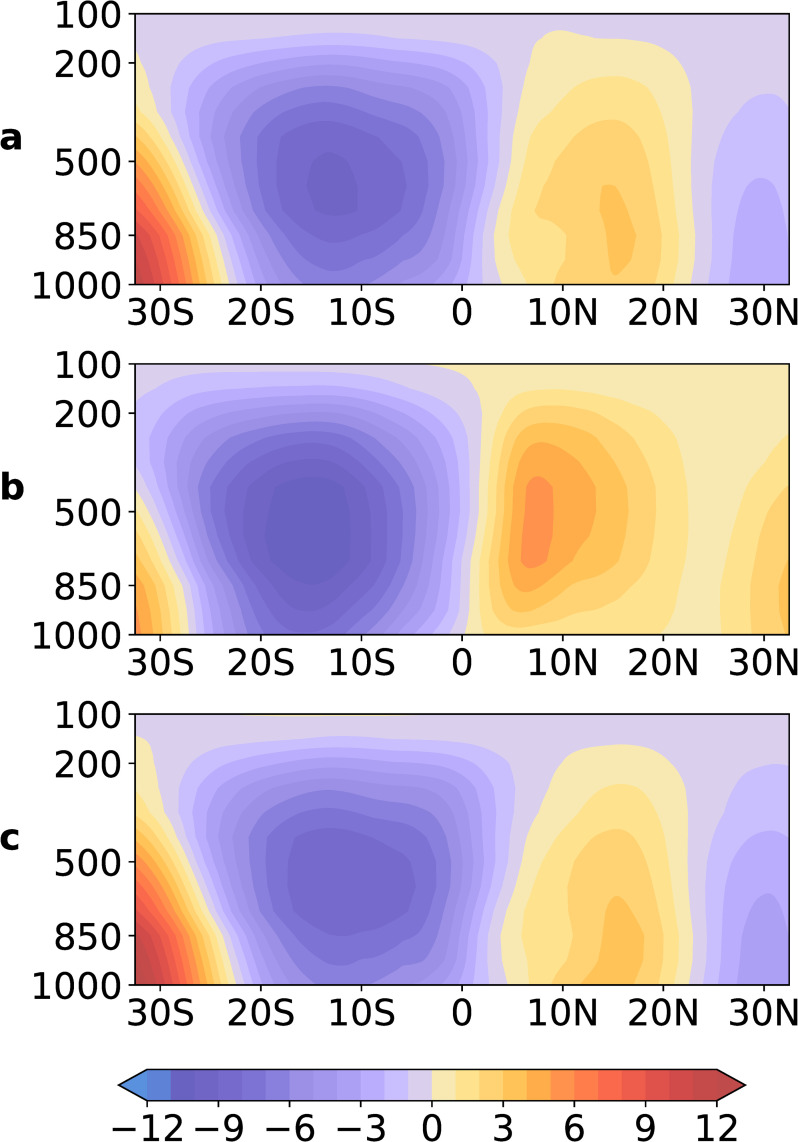
Composite MCA from May to October during the developing years of eight El Niño events: a, composite MCA from May to October during eight El Niño developing years for a total of 48 months; b, without the updraft in the North Pacific; c, with the updraft in the North Pacific. Shaded areas indicate the MSF of MCA (unit: 10^10^ kg s^-1^). The purple regions indicate counter-clockwise movement and the red or yellow areas indicate clockwise movements.

The asymmetry of the MCA mainly occurs from May to October during the El Niño developing year (Fig 2, dev5–dev10, marked in red). To clarify the effect of the updraft on MCA, we composited the MCA from May to October during the developing years of eight El Niño events, and the results are presented in Fig 3. The MCAs in the North and South Pacific show obvious intensified asymmetry. The MCA in the South Pacific was stronger than that in the North Pacific (Fig 3A). We further divided these months into two groups: with and without the updraft. We calculated the average vertical velocity anomaly in the North Pacific (0°–20°N, 140°E–140°W, 10-hPa–1000-hPa). An updraft is considered to be a vertical velocity anomaly of greater than 0, while the absence of an updraft is considered to be a vertical velocity anomaly of less than 0. The results with and without the updraft are shown in Fig 3B and 3C, respectively. The occurrence of an updraft (Fig 3C) causes significantly weaker MCA intensity in the North Pacific than without the updraft (Fig 3B). As the sinking branch of the MCA in the North Pacific is opposite to the local updraft, the updraft effectively weakens the sinking branch. The updraft in the North Pacific therefore weakened the strength of the MCA in the North Pacific. In the following sections, we explore the effects of the updraft further.

### Atmospheric quantities related to the updraft

In the previous section, we connected the updraft to the asymmetry of the MCA. Next, we connect several atmospheric quantities to the updraft, which will extend the connection between TCs and the asymmetry of the MCA. First, we determined the atmospheric quantity that may affect the updraft during El Niño years. We selected two atmospheric quantities that may affect the updraft in the North Pacific: the EWW and NRV.

We used the zonal average wind speed anomaly at 850-hPa in the equatorial region (5°S–5°N, 140°E–140°W) to define EWW (see method). The ONI and EWW belong to the SSTA and the Southern Oscillation of ENSO, respectively. All variables form aspects of ENSO, and the connection between them was recognised as early as 1969 [31]. The EWW can also be regarded as the atmospheric part of ENSO—i.e., the near-surface airflow branch of the Walker circulation—and is driven by the SSTA. According to the definition of relative vorticity in fluid mechanics, the shear vorticity is related to the uneven distribution of the horizontal wind speed along the normal direction of the streamline. When the EWW flows from west to east at the equator, it interacts with the northeast trade wind flowing towards the equator, which increases the wind speed shear and vorticity. The regions on the equatorward side of the trade winds are areas of large-scale surface cyclonic wind shear (large-scale relative vorticity) [32]. These horizontal shear regions are considered to have an important influence on the convergence and upward vertical motion of the airflow [32]. Therefore, we used the regional average relative vorticity in the North Pacific (0°–20°N, 140°E–140°W) to define NRV to explore the relationship between EWW, NRV, and the updraft.

We also considered TCs in order to link the asymmetry of the MCA to TCs. TCs are low-pressure vortices that occur on tropical or subtropical ocean surfaces and are powerful tropical weather systems. Research suggests that the relative vorticity of the lower atmosphere plays an important role in the generation and development of TCs [32]. According to research on the genesis potential (GP) index of TCs, the vorticity anomaly is considered to contribute significantly to the GP of TCs in the Central Pacific [33]. Relative vorticity has an important influence on the TCs generation. Therefore, an enhanced NRV may increase the frequency of TCs. This will extend the connection between TCs and MCA asymmetry.

**Fig 4:**
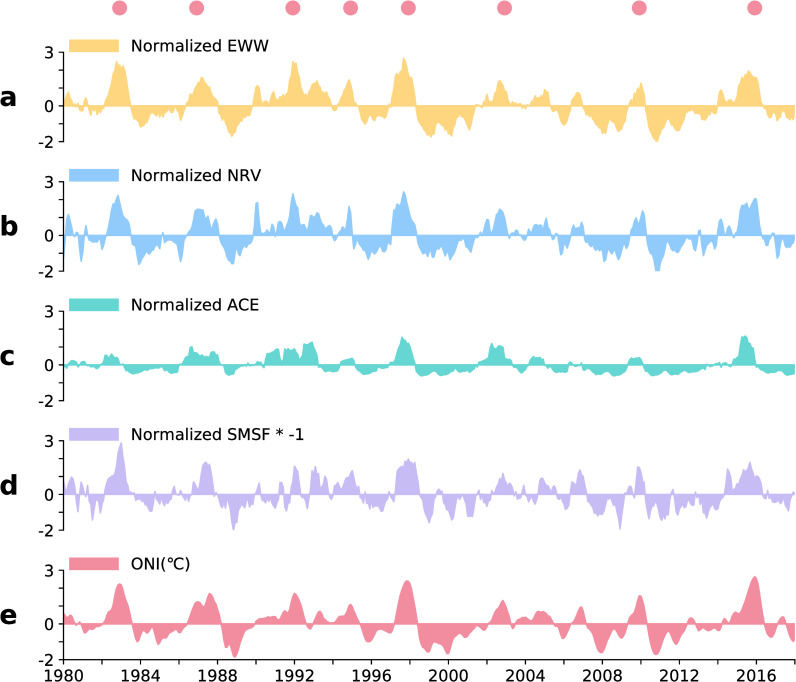
Inter-annual changes in various variables from 1980 to 2018: a, interannual change of EWW; b, interannual change of NRV; c, interannual change of ACE; d, interannual change of MCA; e, interannual change of ONI (°C). All variables except ONI are standardised. The red dots represent the 1982–83, 1986–87, 1991–92, 1994–95, 1997–98, 2002–03, 2009–10, and 2015–16 El Niño events from left to right.

The ACE index is the measure of TCs activity used by NOAA, which reflects the overall intensity and duration of the tropical cyclone activity. The ACE index calculation selects the monthly active TCs in the North Pacific (0°–20°N, 140°E–140°W). We used the MSF to describe the MCA in the South Pacific (SMSF * -1). For ENSO, the SSTA (ONI) in the NINO-3.4 area (5°N–5°S, 120°W–170°W) was used to define its intensity. Except for the ONI, all elements were standardised for better comparison. The inter-annual changes in each element are shown in Fig 4. Each element and ENSO show the same change, which suggests that each element is controlled by ENSO.

**Fig 5:**
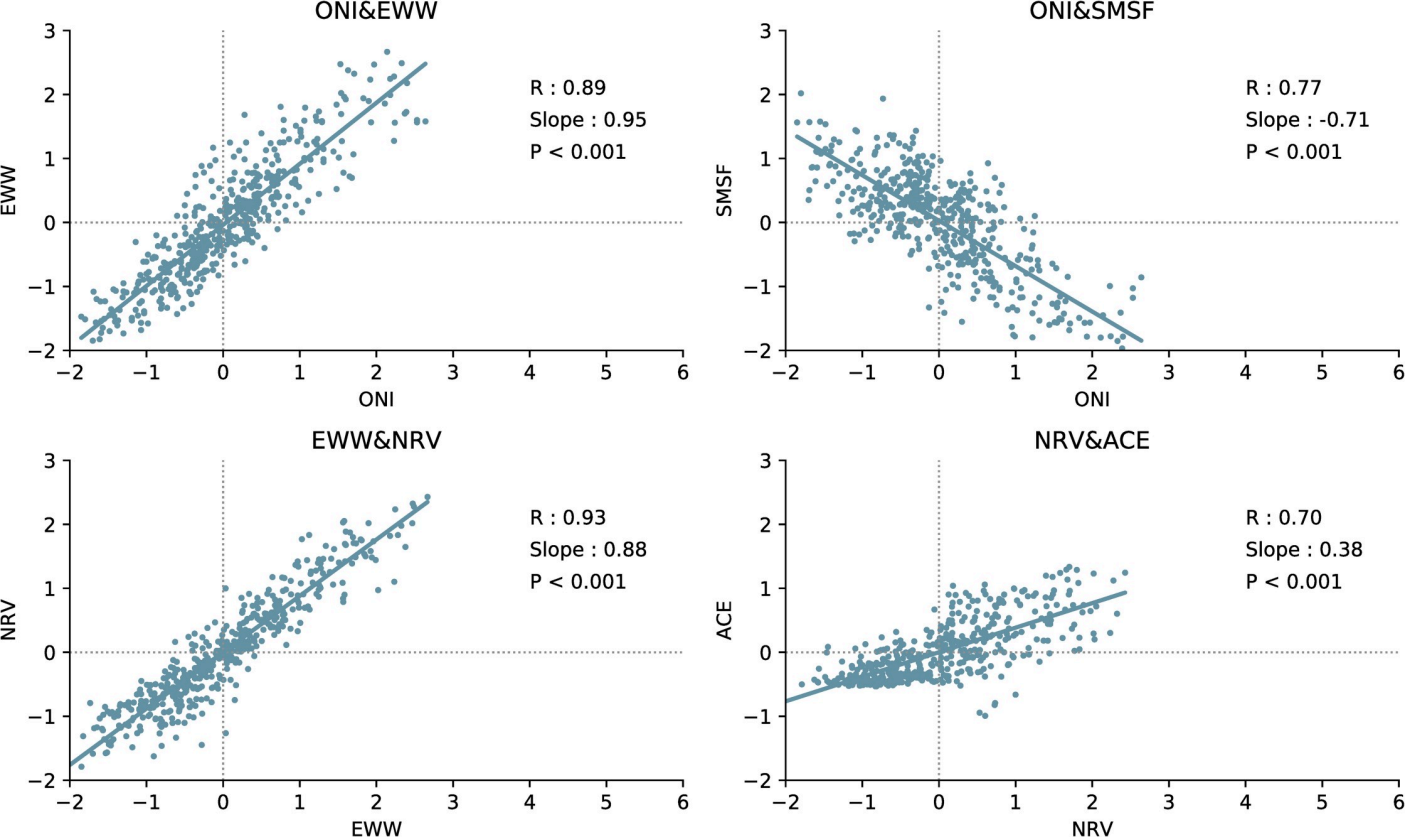
Linear regression between each variable. R: correlation coefficient; Slope: slope of the linear regression equation; P: P value.

The EWW is the near-surface atmospheric branch of the Southern Oscillation of ENSO and is strictly controlled by the SSTA in the Central and eastern Pacific [31]. The correlation coefficient between ONI and EWW was 0.89 (Fig 5).

Research shows that the MCAs between 0° and 30°N in the North Pacific and between 0° and 30°S in the South Pacific are closely related to ENSO events [29]. The MCA in the North and South Pacific during ENSO can be regarded as the direct thermal circulation released by the equatorial SSTA energy [30]. It must therefore be controlled by the SSTA in the Central and eastern Pacific. The correlation coefficient between ONI and SMSF was 0.77 (Fig 5).

According to the definition of relative vorticity in fluid mechanics, the shear vorticity is related to the uneven distribution of the horizontal wind speed along the normal direction of the streamline. When the EWW flows from west to east at the equator, it interacts with the northeast trade wind (flowing to the equator) to form a greater wind speed shear, which enhances vorticity in the North Pacific. Therefore, the EWW may have a positive effect on the relative vorticity anomaly in the North Pacific. The correlation coefficient between EWW and NRV was 0.93 (Fig 5).

El Niño affects the generation and development of TCs by affecting various atmospheric and oceanic conditions [32]. These include vorticity, relative humidity, and vertical wind shear. The effect of these conditions on the generation and development of TCs remains controversial and requires further study. Many studies have found that the relative vorticity of the lower atmosphere plays an important role in the generation and development of TCs [32, 33]. According to research on the GP index of TCs, the vorticity anomaly is considered to contribute significantly to the GP of TCs in the Central Pacific [33]. Therefore, NRV may have an important influence on the generation and development of TCs. The correlation coefficient between NRV and ACE was 0.70 (Fig 5).

In summary, we propose two atmospheric quantities (EWW and NRV) that may affect the updraft. This connection would suggest a link between TCs and MCA asymmetry.

### Combined effect of the equatorial westerly wind and relative vorticity on the updraft

In the previous sections, we found that updrafts can enhance the asymmetry of the MCA in the North and South Pacific, and some atmospheric quantities related to the updraft may contribute to the formation and development of TCs. The updraft controlled by El Niño may become a bridge linking the MCA and TCs. To investigate the combined effects of the EWW and NRV on the updraft, we assessed composite wind, relative vorticity, total precipitation, and the vertical velocity anomaly from May to October during eight El Niño development years.

**Fig 6:**
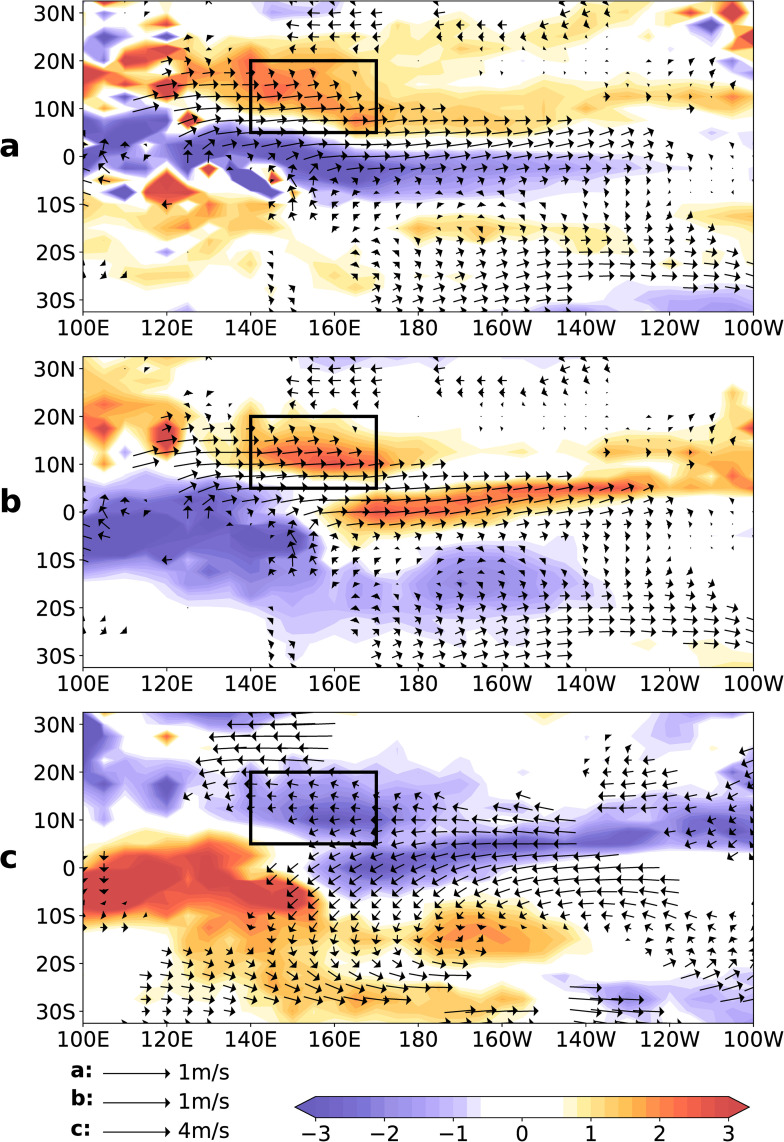
Composite wind speed, relative vorticity, precipitation, and the vertical movement anomaly from May to October during eight El Niño’s developing years: a, the arrow indicates the wind anomaly (m s^-1^) at 850-hPa, and the shaded area indicates the relative vorticity anomaly (5 * 10^−5^ s^-1^) at 850-hPa; b, the arrow indicates the wind anomaly (m s^-1^) at 850-hPa, and the shaded area indicates the total precipitation anomaly (mm); c, the arrow indicates the wind anomaly (m s^-1^) at 200-hPa, and the shaded area indicates the vertical movement anomaly (10^−2^ Pa s^-1^) at 500-hPa. Arrows indicates a significance level of > 95% using the Student’s t-test. The black rectangle (5°N–20°N, 140°E–170°E) represents the monsoon trough region related to relative vorticity.

The regions on the equatorward side of the trade winds are areas of large-scale surface cyclonic wind shear (large-scale relative vorticity) [32]. The EWW interacts with the northeast trade wind to form greater wind speed shear, resulting in a greater relative vorticity anomaly in the North Pacific during El Niño years. The results are presented in Fig 6A, where the arrow indicates a wind anomaly at 850-hPa, and the shaded area indicates the relative vorticity anomaly at 850-hPa. The EWW and northeast trade winds produce positive shear, forming a greater relative vorticity anomaly in the black rectangle (5°N–20°N, 140°E–170°E) (Fig 6A). The relative vorticity anomaly in this region is closely related to the MT. Research has shown that MT in the western North Pacific, defined by the average meridional (5°N–20°N) positive relative vorticity at 850-hPa from July to November each year, provides useful information and allows for the inter-annual comparison with ENSO [34]. Specifically, studies have shown that the interannual variations in MT is a response to ENSO [35]. An El Niño event increases the relative vorticity over the western central tropical North Pacific, resulting in an eastward extension of the MT [35]. A strong MT expands south-eastward to near 170°E, whereas a weak MT is confined to the west of 140°E [36]. This finding is consistent with our results. Due to the link between NRV and ENSO, the EWW may be the reason for the increased relative vorticity and eastward extension of the MT. Therefore, the black rectangle is defined as the MT region related to relative vorticity (MT-RV), which will be referred to as the MT-RV in subsequent discussions.

The MT is defined as the region between the low-level equatorial westerlies on its equatorward side and the low-level trade wind easterlies on its poleward side. It is characterised by a local, zonally elongated sea level pressure minimum and enhanced rainfall [37]. Consistent with previous research, the MT-RV is also characterised by enhanced rainfall. The results are presented in Fig 6B, where the arrows indicate the wind anomaly at 850-hPa, and the shaded areas indicate the total precipitation anomaly. We observed higher rainfall in the MT-RV (Fig 6B), and an increase in rainfall led to an increase in the release of latent heat at high altitudes. The high altitude is heated by the release of latent heat, accompanied by an increase in the local ascending motion. The results are shown in Fig 6C, where the arrow indicates the wind anomaly at 200-hPa, and the shaded area indicates the vertical movement anomaly at 500-hPa. We observed a strong updraft in the MT-RV (Fig 6C).

The same result was also observed for the monthly changes. We assessed the composite meridional mean (5°N–20°N) monthly sea surface temperature, the EWW, relative vorticity, total precipitation, and vertical velocity anomaly from May to October during the development of eight El Niño events.

**Fig 7:**
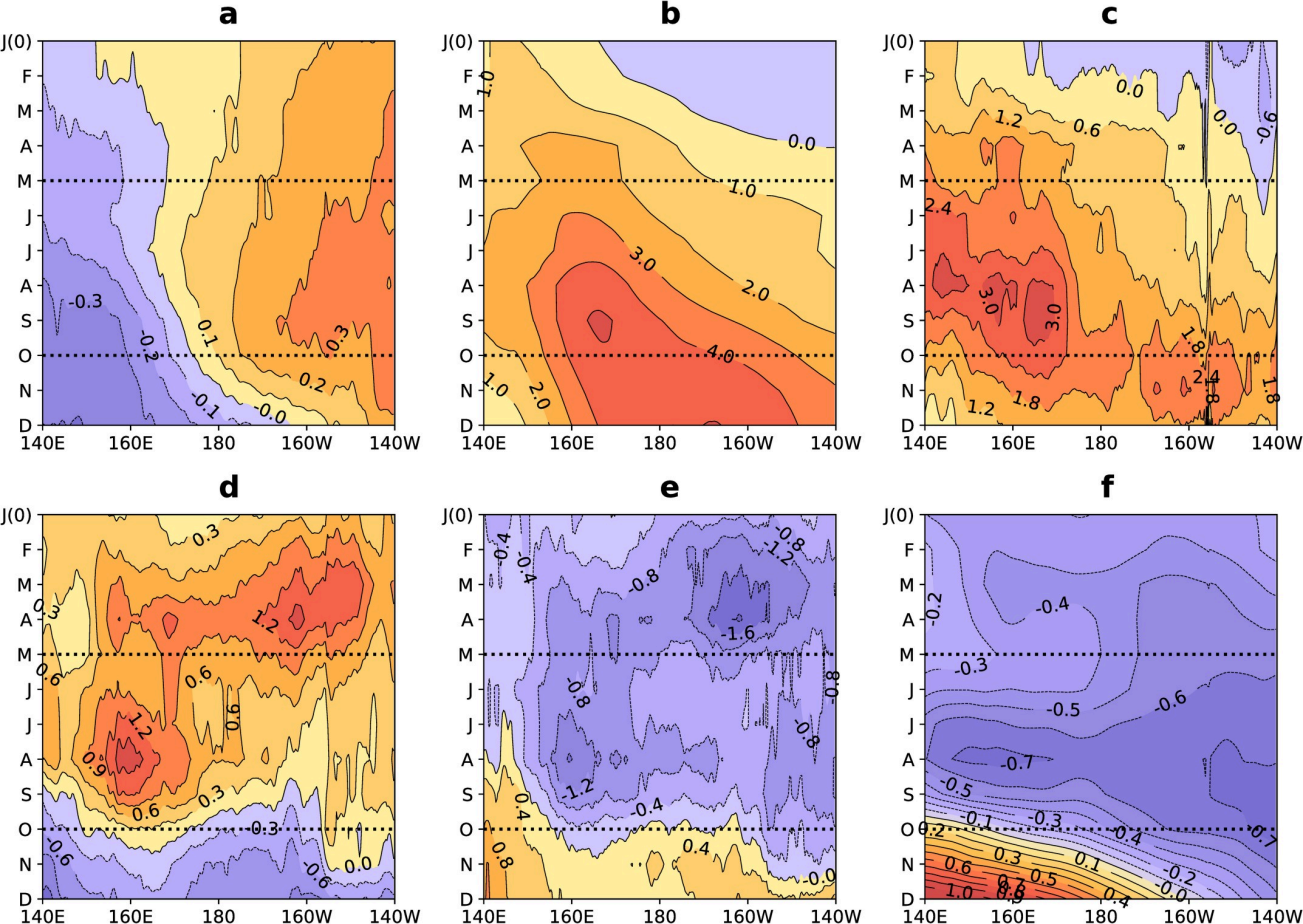
Composite meridional mean (5°N–20°N) monthly (a) sea surface temperature anomaly, (b) equatorial westerly wind anomaly, (c) relative vorticity anomaly, (d) total precipitation anomaly, (e) vertical velocity anomaly, and (f) sea level pressure anomaly during eight El Niño developing years: a, the shaded area indicates the meridional mean (5°N–20°N) sea surface temperature anomaly (°C); b, same as in a, but for the equatorial westerly wind anomaly (m s^-1^) at 850-hPa; c, same as in a, but for the relative vorticity anomaly (10^−6^ s^-1^) at 850-hPa; d, same as in a, but for the total precipitation anomaly (mm); e, same as in a, but for the vertical velocity anomaly (10^−2^ Pa s^-1^) at 500-hPa; f, same as in a, but for sea level pressure anomaly (hPa). J (0)-D indicates January to December during an El Niño developing year. The black dotted lines represent May to October.

First, we considered whether the MT-RV has similar characteristics to that of the MT. The MT has been found to be related to the sea level pressure minimum and enhanced rainfall [37]. The relative vorticity anomaly (RVA) at 850-hPa, total precipitation anomaly (TPA), and sea level pressure anomaly (SLPA) are shown in Fig 7C, 7D and 7F, respectively. The results show that a strong positive RVA, strong positive TPA, and strong negative SLPA correspond to the MT-RV (140°E–170°E) from May to October (Fig 7C, 7D and 7F). This further confirms that MT-RV has MT characteristics.

Next, we considered the vertical velocity anomaly (VVA) from the perspective of the SSTA. The VVA and SSTA are shown in Fig 7A and 7E, respectively. We observed two strong updraft regions from May to October: (1) near 160°E and (2) near 150°W (Fig 7E). The strong updraft region near 150°W corresponds to a positive SSTA (Fig 7A and 7E), while the strong updraft region near 160°E corresponds to a negative SSTA (Fig 7A and 7E). However, a negative SSTA cannot explain this strong updraft region, which is located in the MT-RV. We therefore explain the strong updraft region from the perspective of the MT-RV.

The EWW anomaly is considered to be an important component. EWW is shown in Fig 7B. The EWW increased from May to October, and a strong positive EWW appeared in the MT-RV (Fig 7B). The EWW and the northeast trade winds produce a positive shear, forming a strong positive RVA in the MT-RV (Fig 7C), which explains the close link between EWW and NRV (correlation coefficient = 0.83, Fig 5). A strong positive RVA corresponds to a strong positive TPA and a strong negative VVA in the MT-RV (Fig 7C–7E). This is because a strong positive RVA is accompanied by the MT, which enhances TPA and the updraft in the MT-RV. Therefore, EWW and MT-RV are closely related to the enhancement of the updraft.

By building the connection between MT and NRV, we linked the EWW, NRV, and the updraft. Next, we demonstrate how the MT-RV acts as a bridge connecting TCs.

### Connection between relative vorticity and tropical cyclones

The relationship between relative vorticity and TCs can also be explained by the MT-RV. Research has shown that the MT in the western North Pacific can largely determine the area of mean TCs formation by providing favourable thermodynamic and dynamic large-scale conditions for TCs formation, such as cyclonic relative vorticity, a moist mid-level troposphere, and weak vertical wind shear [38, 39]. The formation conditions [38], formation region [34], and interannual [40] and decadal [41] changes in TCs are all closely related to MT. Therefore, TCs activity in the western North Pacific is closely related to MT. The interannual variation of MT has been found to be a response to ENSO [35]. An El Niño event causes an increase in relative vorticity over the western central tropical North Pacific and thus an eastward extension of the MT [35]. The formation of enhanced tropical storms (a class of TCs) in the south-eastern quadrant (5°N–17°N, 140°E–180°E) of the western North Pacific has been attributed to increases in the low-level shear vorticity generated by El Niño–induced equatorial westerlies [40]. Therefore, the enhanced eastward extension of the MT may be related to the strengthening of TCs activities in the south-eastern quadrant of the western North Pacific. We therefore used the MT-RV to explain the relationship between TCs and relative vorticity.

**Fig 8:**
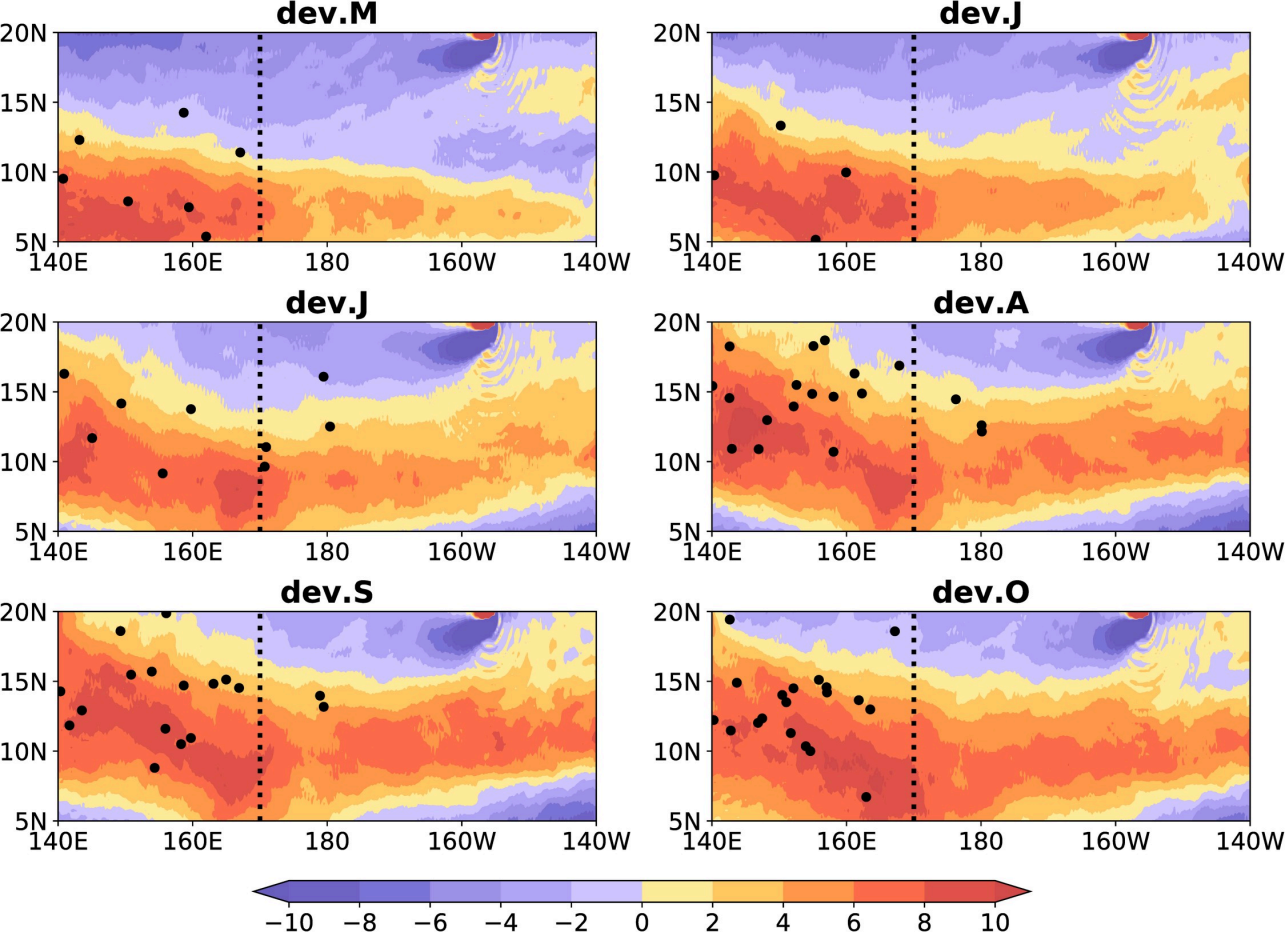
Location of monsoon trough and tropical cyclone. Shaded area indicates the composite monthly relative vorticity (10^−6^ s^-1^) at 850-hPa from May to October during eight El Niño developing years. The monsoon trough related to relative vorticity is the region between 140°E–170°E and with values greater than 0. The black dotted line represents 170°E. The black dots indicate the locations of tropical cyclones. Title dev. M-O indicates the period from May to October during El Niño developing years.

**Fig 9:**
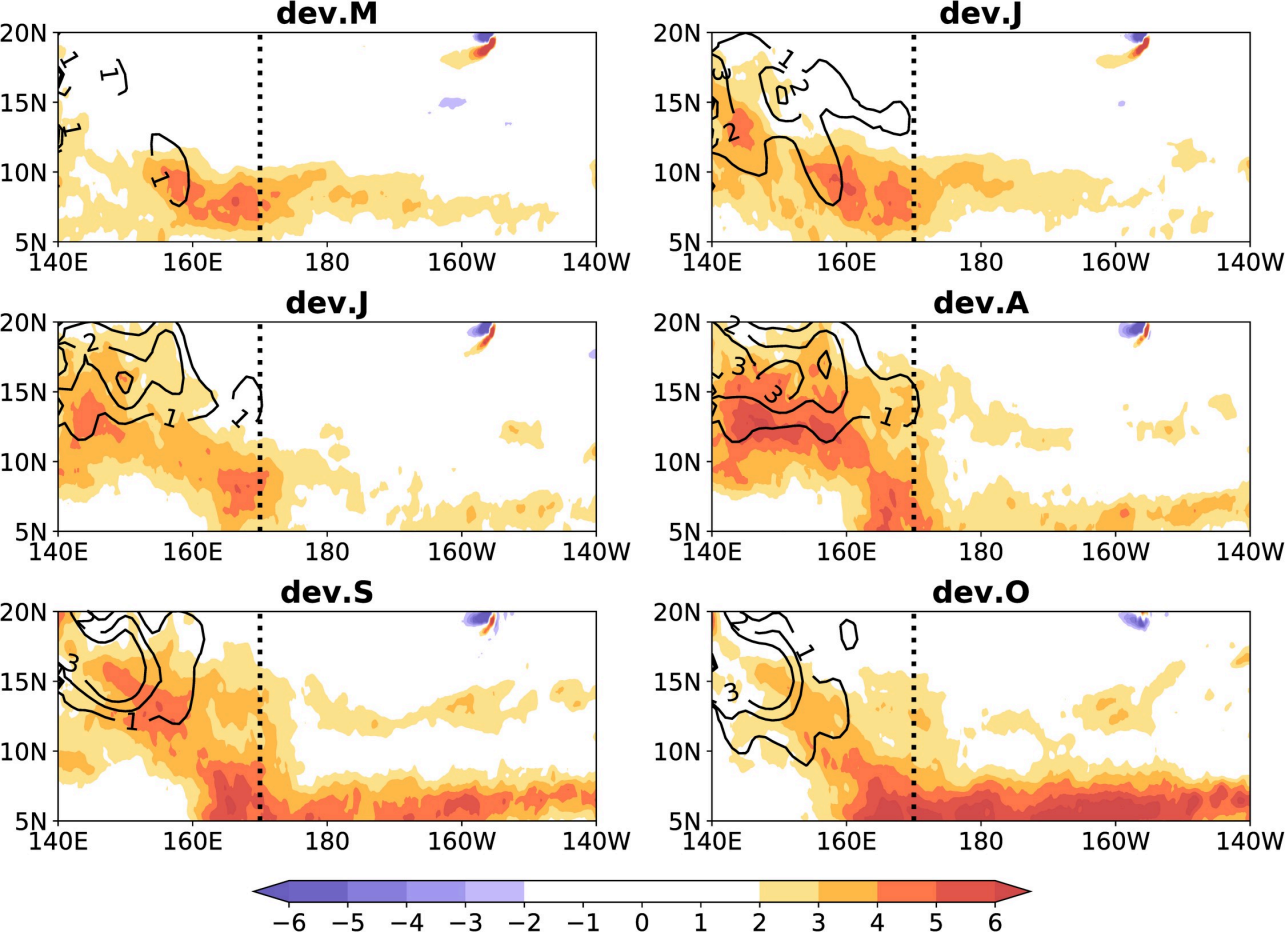
Link between the eastward extension of the monsoon trough and tropical cyclone activity. The shaded area indicates the composite monthly relative vorticity anomaly (10^−6^ s^-1^) at 850-hPa from May to October during eight El Niño developing years. The black dotted line indicates 170°E, and the contour indicates the ACE anomaly (10^2^ m^2^ s^-2^) of tropical cyclones. Title dev. M-O indicates the period from May to October during El Niño developing years.

We studied the composite monthly relative vorticity at 850-hPa and the TCs from May to October during eight El Niño events, and the results are presented in Fig 8. A relative vorticity of > 0 in the MT-RV region is considered to be the MT. Almost all TCs are generated in the MT in the MT-RV region (Fig 8). The seasonal north-south movement of the TCs position was consistent with the MT (Fig 8), which is consistent with previous research [34]. We then investigated whether the relative vorticity anomaly is consistent with the changes in the ACE anomaly of TCs (TCs-ACE). We investigated the composite monthly RVA at 850-hPa and the TCs-ACE anomalies from May to October during the eight El Niño development years, and the results are presented in Fig 9. The shaded area indicates the RVA, and the contour indicates the TCs-ACE anomaly. The TCs-ACE anomaly was consistent with the change in the RVA in the MT-RV region (Fig 9). In the MT-RV region, the TCs-ACE anomaly increases eastward as the RVA increases, and retreats westward as the relative vorticity anomaly decreases (Fig 9). Therefore, the activity of the TCs is related to the relative vorticity in the MT-RV region.

The physical mechanism underlying this association is unclear. Some research shows that increases in TCs activity is attributed to increases in weather-scale disturbances associated with MT in the south-eastern quadrant (5°N–17°N, 140°E–180°E) of the western North Pacific [35]. Synoptic-scale disturbances have been proposed as a possible forcing mechanism for tropical cyclogenesis [42]. An idealised model showed that a tropical disturbance tends to develop over the eastern part of the western North Pacific near 150°E–160°E when the MT extends eastward [36]. Based on previous research, the physical mechanism of the connection remains to be explored. However, the connection between TCs and MTs may contribute to TCs forecasting.

### Asymmetry of the meridional circulation anomaly during each El Niño event

Our research showed that the updraft may be affected by the combined effects of the EWW, NRV, and a warm ocean surface. The background of the relative vorticity and MT is conducive to the generation and development of TCs. Each El Niño event was studied individually.

**Fig 10:**
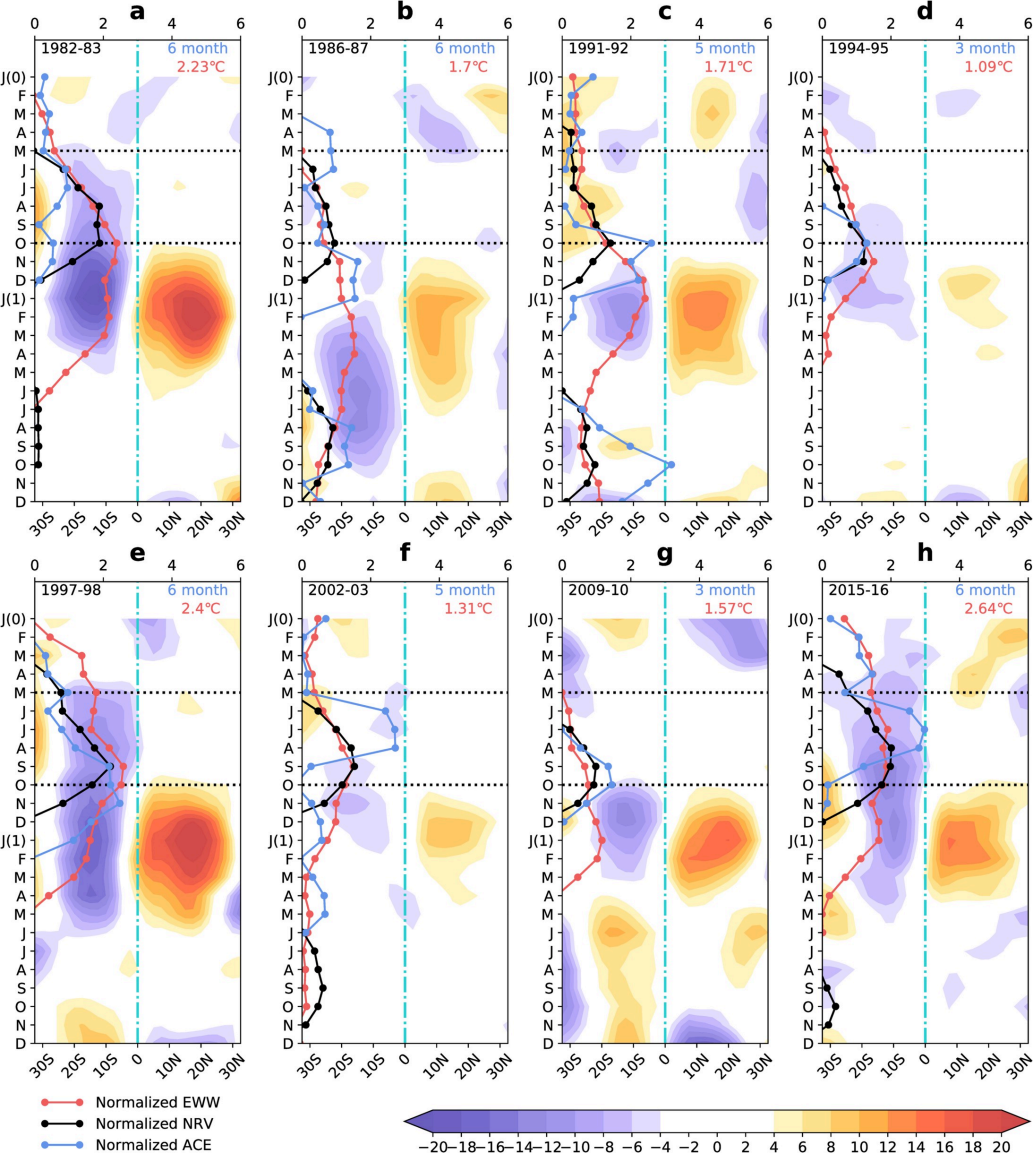
Changes in the elements during eight El Niño events. The shaded area indicates the MSF of MCA (unit: 10^10^ kg s^-1^). The purple area indicates counter-clockwise movement and the red or yellow area indicates clockwise movement. The red line indicates the normalised EWW, the black line indicates the normalised NRV, and the blue line indicates the normalised ACE. The blue numbers in the upper right indicate the months when the ACE was positive from May to October during the developing year. The red numbers in the upper right are the maximum that the ONI can reach during El Niño years. The black numbers in the upper left are each El Niño year. J (0)-D indicates January to December during El Niño developing years. J (1)-D indicates January to December during El Niño decaying years. The black dotted line indicates May to October. The blue dotted line indicates 0°.

The results are presented in Fig 10. We found that the asymmetry of the MCA from May to October during eight El Niño events was stronger during intensified El Niño years. For example, ONI reached a maximum of 2.23°C (Fig 10A), 2.4°C (Fig 10E), and 2.64°C (Fig 10H) in 1982–83, 1997–98, and 2015–16, respectively. During three strong El Niño developing years, we observed a continuously strong MCA over time in the South Pacific but not in the North Pacific from May to October. A possible reason may be due to the rapid development of high SSTAs during strong El Niño events, which leads to a stronger MCA. The MCA in the South Pacific responds to the rapid SSTA development, resulting in continuous and powerful MCAs in the South Pacific in our time series. Accompanied by strong EWW, the NRV and MT were also stronger during intensified El Niño events. The MT related to relative vorticity strengthens the updraft and further blocks the development of the MCA in the North Pacific. The intensity of the SSTA was weak and developed more slowly during five weak El Niño events (1986–87, 1991–92, 1994–95, 2002–03, 2009–10) (Fig 10B–10D, 10F and 10G). The MCA in the South Pacific responds to the slow development of the SSTA. Therefore, it is difficult to observe a continuous and strong MCA in the South Pacific in our time series. The North Pacific does not show a continuously strong MCA, regardless of whether the El Niño event was strong or weak. A weak El Niño leads to a weak SSTA that develops slowly. This observation is similar to the MCA in the South Pacific, and no continuous and intensified MCA was observed in the time series. The SSTA was strong and developed rapidly from May to October during three strong El Niño years (Fig 10A, 10E and 10H). The EWW responds to the rapid development of SSTA, which is conducive to positive NRV, MT, and TCs activities. Both NRV and ACE were strongly positive during three strong El Niño developing years (Fig 10A, 10E and 10H). ACE was also positive in a higher number of months. For example, the ACE was positive in six months during 1982, 1997, and 2015. Moreover, NRV and ACE were closely related to the MCA asymmetry.

## Conclusion

Our research mainly focused on the asymmetry of the meridional circulation anomaly (MCA) in the Central Pacific (30°S–30°N, 140°E–140°W) from May to October during eight El Niño events. We found that the asymmetry of the MCA may be enhanced by the updraft anomaly in the North Pacific (0°–20°N, 140°E–140°W). The updraft anomaly was strengthened by the combined effects of the positive equatorial westerly wind anomaly, positive relative vorticity anomaly, and warm ocean surfaces. Moreover, the background of the updraft anomaly, positive relative vorticity anomaly, and monsoon trough (MT), which are controlled by El Niño in the MT-RV region (5°N–20°N, 140°E–170°E), may be conducive to the generation and development of tropical cyclones (TCs).

Our results show that the updraft anomaly may enhance MCA asymmetry. TCs are connected to the updraft anomaly by the relative vorticity related to the MT in the MT-RV region. The updraft controlled by El Niño may become a bridge linking the MCA and TCs. Therefore, we propose a possible link between TCs and the asymmetry of MCA, however the proof of the physical mechanism is still lack. For instance, the definition of MT is vague and the physical mechanism concerning the formation and development of TCs is lack. In addition, the theory that relative vorticity can promote the generation and development of TCs is still controversial because large-scale environmental factors promoting the generation and development of TCs still lack a clear physical mechanism. Therefore, it may be possible that more TCs would increase the relative vorticity. Moreover, the connection between MT and TCs is more based on statistical research. To further confirm our findings, future studies should use the model to further verify the physical mechanisms.
